# Male involvement in the maternal health care system: implication towards decreasing the high burden of maternal mortality

**DOI:** 10.1186/s12884-018-2139-9

**Published:** 2018-12-14

**Authors:** Amanual Getnet Mersha

**Affiliations:** Department of Gynecology and Obstetrics, School of Medicine, College of Medicine and Health Sciences, University of Gondar, P.O. Box: 196, Gondar, Ethiopia

**Keywords:** Ethiopia, Birth preparedness, Complication readiness

## Abstract

**Background:**

One of the essential components of antenatal care (ANC) is birth preparedness and complication readiness (BP/CR). Strengthening BP/CR measures is one of the principal strategies to reduce maternal mortality and morbidity. The current study aimed at determining the level of men’s knowledge about obstetric danger signs, and their involvement in BP/CR among community of Northwest Ethiopia**.**

**Method:**

A cross-sectional community based survey was conducted in Northwest Ethiopia from May 2016 to July 2016. Data was analyzed by the Statistical Package for the Social Sciences software Version 21.0 for Windows. Participants’ socio-demographic characteristics, knowledge of obstetric danger signs, and level of involvement in BP/CR were described using frequencies and percentages. Bivariate and multivariable logistic regressions were employed to explore the associated factors and *P*-value of 0.05 was used as a cut-off point to declare significant association.

**Result:**

From 856 men who were invited for the study, 824 men agreed for the interview giving a response rate of 96.2%. Half of the men stated one danger sign that may occur during pregnancy 407(49.4%); one third during delivery 271(32.9%); and 213(25.8%) during postpartum period. Among all participants, 256(31.1%) had not made any preparations; 363(44.1%) made one step; 116(14.1%) made two steps; 82(9.9%) made three steps; 5(0.6%) made four steps; 2(0.24%) made five steps; and no one made all the birth preparation steps during the birth of their last child. BP/CR was significantly association with knowledge of at least one danger sign during pregnancy (AOR = 3.3, 95% CI: 3.1, 3.9); during delivery (AOR = 2.2, 95% CI: 1.1, 2.8); and post partum period (AOR = 1.8, 95% CI: 1.1, 2.4). Furthermore, BP/CR was found to be positively associated with being married, completing college education, escorting wife to antenatal care, and urban residence.

**Conclusions:**

Men’s level of knowledge about obstetric danger signs, and their involvement in BP/CR was found to be very poor. Considering the importance of male involvement in the maternal health care, it is recommended to advocate policies and strategies that can improve awareness of men and enhance their engagement in the maternal care.

**Electronic supplementary material:**

The online version of this article (10.1186/s12884-018-2139-9) contains supplementary material, which is available to authorized users.

## Background

Globally, an estimated number of 303,000 maternal deaths occur annually from causes related to pregnancy and childbirth. Around 99% of these deaths occur in developing countries and sub-Saharan African accounts for almost half of the maternal deaths (44%) [1, 2]. Maternal death may occur from complications that may occur while a woman is pregnant, during labor or after delivery. Preparing for birth and related complications ahead of time markedly reduce the number of women dying from such preventable causes [3].

One of the essential components of antenatal care (ANC) is birth preparedness and complication readiness (BP/CR). Birth preparedness and complication readiness (BP/CR) includes detection of danger signs, a plan for a birth attendant, a plan for the place of delivery, preparing potential blood donor and saving money for transport or other [4]. Some of the obstetric complications such as hemorrhage are difficult to precisely predict which mother will develop the complications, therefore every pregnant woman and spouse should make the necessary birth plans [5]. After the International Conference on Population and Development (ICPD) and 4th World Conference on Women held at Cairo [6] and Beijing [7] respectively, men’s involvement in maternal health care system is being advocated. Studies had shown the helpful impact of male participation in maternal health in developing countries by improving maternal access to antenatal and postnatal services [8, 9].

The 2015 World Health Organization (WHO) recommendation on maternal and newborn health promotion interventions included active involvement of men during pregnancy, child birth and post partum period as an effective intervention to improve maternal as well as newborn health outcomes. However, male involvement is recommended provided only that women’s autonomy in making their own decisions is respected [8].

A systematic review that included 13 studies aimed at assessing the effect of male partner involvement in low and middle income countries as an intervention to improve maternal health outcomes was published in 2018. The review demonstrated that male engagement as an intervention improves antenatal care utilization, postpartum care utilization, delivery at health facilities, child birth by a skilled attendant, and birth preparation and complication readiness [9].

Social norms, beliefs and values affect the types and extent of support that a pregnant woman can receive from her husband or other family members. For instance, a study conducted in Bangladesh showed low level of facility delivery service utilization among women whose husbands believed childbirth is a natural physiologic process that does not require a medical care; and women whose husbands were pressurized by other family members about appropriate place of delivery [10].

In societies where social norms, beliefs and values weaken women’s rights, men have social as well as economical supremacy over their partners. Hence, in such patriarchal societies men make a decision regarding sexual affairs, family size, women’s access to economic resources and health care service utilization. Achieving sustainable development goals (SDGs) requires alleviating gender based inequalities as well as improving male partner participation in the maternal health care system [11]. Male involvement should be continuously monitored so that it will not worsen gender inequality and affect women’s reproductive rights [8].

A study conducted in Oromia regional state of Ethiopia showed that 89% of males were involved in deciding home as their spouses place of deliver [12]. In Patriarchal societies social norms, values and low attitude towards empowering women affects a women’s level of maternal health service utilization. Hence, a careful engagement of male partners in the maternal health service in such communities may be an effective strategy to improve maternal health service utilization and reduce maternal morbidity as well as mortality. It is essential to assess men’s current level of awareness and involvement in the maternal health care system in order to plan an effective intervention strategy to improve their involvement. Therefore, the current study aimed at assessing men’s level of knowledge concerning obstetric danger signs, their level of birth preparedness and complication readiness (BP/CR) in Northwest Ethiopia.

## Methods

### Study design and setting

A cross-sectional descriptive community survey was conducted in North Gondar Zone, Northwest Ethiopia from May 3, 2016 to July 5, 2016. This zone has 19 districts; the zone has a total population of 2,903,165, of this 14.1% being urban residents.

### Sample size and sampling technique

The sample size was calculated by using single population proportion formula by taking 5% desired precision, 95% confidence interval, assuming 42% men awareness of danger signs [21], design effect of 2 for cluster sampling and 10% non response rate, which results in sample size of 710. A multi stage cluster sampling procedure was employed to select four districts out of 19 districts of North Gondar zone. Firstly four districts, three rural districts and one urban district were randomly selected. All households within the catchment areas having men whose wife had given birth within the last 2 years were invited to interview. A total of 856 men were invited to participate in the study.

### Data collection and management

A self administered questionnaire was adopted for our setting from Johns Hopkins Program for International Education in Gynecology and Obstetrics (JHPIEGO) [13]. The questionnaire has three sections: The first section is regarding socio demographic characteristics of the participants. The second section assesses participant’s knowledge of obstetric danger signs during pregnancy, labor and post partum periods. The final section assesses birth preparedness and complication readiness among participants during the birth of their last baby. The data collection process had taken 2 months (from May 3, 2016 to July 5, 2016). Data was collected by five properly trained research assistants with previous experiences in survey data collection. So as to maintain the quality of the data the researcher reviewed collected data daily and sent feedbacks to the data collectors continuously. BP/CR practice was considered “well prepared” for BP/CR when at least three of the following six practices were reported to have been made in the birth of the last baby: prepared birth kit, identified a skilled attendant, saved money, knew where to go in case of emergency, contacted a blood donor in advance and prepared transportation in advance. Men who reported less than half of the above stated steps were considered to be less prepared. If at least three steps were reported as having been made, the respondent was labeled as well prepared. The three out six was chosen because previous studies have used 50% and above to determine who was well prepared [14]. The questionnaire, originally written in English, was translated to local language (Amharic) and back to English in order to ensure that the translated version gives the proper meaning. The content validity of the questionnaire was confirmed by local experts, including reproductive health experts. (Additional file 1) The questionnaire was pretested on 5% of the sample size prior to the real data collection, which was excluded from the final study. Considering the high burden of puerperal sepsis in this community, which is common in the first 10 days of postpartum period and accounts for 13% of maternal deaths in Ethiopia [15], The time frame of first 2 days in the questionnaire used by Johns Hopkins Program for International Education in Gynecology and Obstetrics (JHPIEGO) was amended to cover maternal health problems in the first 10 days of the post partum period. Once data were collected, each questionnaire was checked for completeness.

### Operational definitions

#### Male partner

Male who has/had a spouse by means of formal marriage or informal union.

#### Birth preparedness and complication readiness (BP/CR)

Is a process of planning for normal birth and anticipating the actions needed in cases of an emergency.

#### Level of birth preparedness and complication readiness (BP/CR)

The steps made to have a normal birth outcome during the previous child birth.

#### Well prepared for birth

If at least three of the following steps have been made in the childbirth process of the last baby: prepared birth kit, identified a skilled attendant, saved money, knew where to go in cases of emergency, contacted a blood donor in advance and prepared transportation in advance.

### Statistical analysis

The final data collection tool was checked for completeness, and responses were entered into and analyzed by the Statistical Package for the Social Sciences software Version 21.0 for Windows. Participants’ socio-demographic characteristics, knowledge of obstetric danger signs, and level of BP/CR were described using frequencies and percentages in tables. Bivariate logistic regression was conducted to explore factors associated with BP/CR and factors that were found to have a *p* value of less than 0.2 were entered in to multivariable logistic regression. To declare significant association *P*-value 0.05 was used as a cut-off point and results expressed by using odds ratio (OR) with 95% confidence interval (CI).

### Ethical considerations

This study was approved by the Institutional review board of University of Gondar. Informed written consent was obtained from each participant before starting the interview and participants were also told their right to stop the interview at anytime. The participants were also told that participation is based on their willingness. The obtained information was kept anonymous and recorded in such a way that the respondent could never be known.

## Results

### Socio-demographic characteristics of participants and their spouses

From 856 men who were invited for participation, 824 men agreed to the interview giving a response rate of 96.2%. A total of 721(87.5%) of the participants were found to be married and half of them have 3–4 children 403(48.9%). One third of the respondents 288(34.9%) did not go to school or did not complete their primary education. Participants from the rural areas accounts for the majority of respondents 637(77.3%). Participants who had escorted their wives to ANC follow up in previous pregnancy accounted for 334(40.5%) of the total participants and a quarter 201(24.4%) of the participants’ spouses had delivered in a health facility. A total of 118(14.3%) participants’ spouses had obstetric complication in their last pregnancy. (Table 1).

**Table 1:** Socio-demographic characteristics of men and spouse’s obstetric characteristics

Characteristic	N (%)
Age	
18–24	154(18.7%)
25–34	198(24%)
35–44	353(42.8%)
> 44	119(14.4%)
Marital status	
Single/divorced/widowed	103(12.5%)
Married	721(87.5%)
Number of children	
1–2	173(21%)
3–4	403(48.9%)
More than 4	248(30.1%)
Education	
No education	150(18.2%)
Primary Incomplete	138(16.7%)
Primary Complete	311(37.7%)
Secondary	124(15%)
College and above	101(12.3%)
Residence	
Rural	637(77.3%)
Urban	187(22.7%)
Escorted wife to ANC previous pregnancy	
Yes	334(40.5%)
No	490(59.5%)
Obstetric complication in previous pregnancy	
Yes	118(14.3%)
No	706(85.7%)
Place of delivery previous pregnancy	
Home	623(75.6%)
Health facility	201(24.4%)

### Knowledge of obstetric danger signs

Half of the participants had stated at least one danger sign during pregnancy 407(49.4%); 271(32.9%) of the participants stated at least one danger sign during delivery 271(32.9%) and fewer, 213(25.8%), of the participants stated at least one danger sign during the postpartum period. The commonest mentioned danger sign during pregnancy was high fever 105(12.7%) and excessive vaginal bleeding accounts for the majority of responses during childbirth as well as during the post partum period. 85(10.3%) participants mentioned prolonged labor as a danger sign. There were also other signs mentioned which were not stated in the table such as anemia, dizziness, palpitation that accounted for 10.1% during pregnancy, 3.2% during delivery and 2.7% during postpartum period. (Table 2).

**Table 2:** Men’s knowledge of danger signs during pregnancy, labor and postpartum

Obstetric danger sign	N (%)
During Pregnancy	
High fever	105(12.7%)
Severe abdominal pain	97(11.8%)
Excessive vaginal bleeding	94(11.4%)
Abnormal body movements	61(7.4%)
Severe headache	70(8.5%)
Swollen hands/face	59(7.2%)
Loss of consciousness	33(4%)
Blurred vision	13(1.6%)
Knowledge of at least one sign	407(49.4%)
During Childbirth	
Excessive vaginal bleeding	102(12.4%)
Abnormal body movements	54(6.5%)
Retained placenta	37(4.5%)
High fever	98(11.9%)
Prolonged labor	85(10.3%)
Severe headache	64(7.8%)
Loss of consciousness	29(3.5%)
Knowledge of at least one sign	271(32.9%)
During postpartum	
Excessive vaginal bleeding	105(12.7%)
High fever	99(12%)
Abnormal body movements	56(6.8%)
Loss of consciousness	31(3.8%)
Foul smelling discharge	74(8.9%)
Severe headache	78(9.5%)
Knowledge at least one sign	213(25.8%)

Multiple responses possible

### Level of BP/CR in spouses’ previous pregnancy

**Fig. 1:**
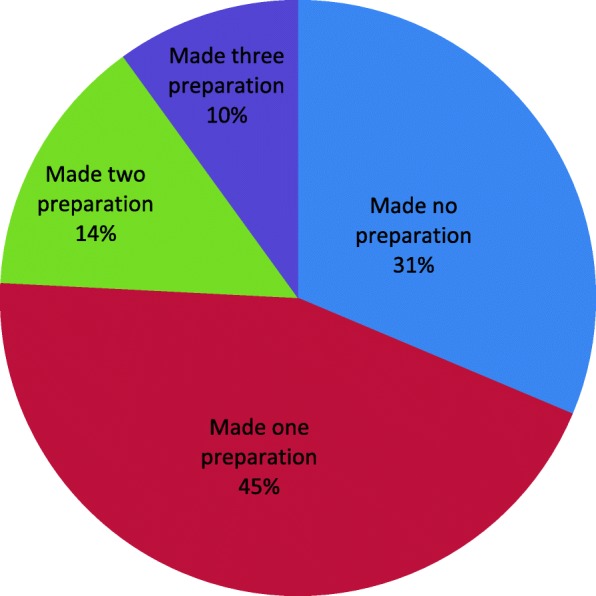
Number of Preparations Made By Participants

As illustrated in table three, the most common preparations made during the birth of their last child were getting birth kit 309(37.5%) followed by saving money 218(26.5%). (Table 3) Furthermore, 91(11%) of the participants had arranged transportation, 25(3%) had identified which health facility to visit in case of emergency, 67(8.1%) had identified a skilled birth attendant, 3(0.4%) prepared possible blood donor during the birth of their last baby. Two hundred and fifty six men (31.1%) had not done any preparations; 363(44.1%) had made one of the six birth preparing steps assessed; 116(14.1%) made two preparations; 82(9.9%) made three preparations; 5(0.6%) made four preparations; 2(0.24%) had made five preparations; furthermore, no one made all the six preparations assessed in the current study. (Fig. 1).

**Table 3:** BP/CR among men in previous pregnancy

BP/CR	N (%)
Identified birth kit	309(37.5%)
Saved money	218(26.5%)
Identified transport	91(11%)
Identified a blood donor in advance	3(0.4%)
Identified where to go for emergency	25(3%)
Identified skilled attendant	67(8.1%)
Made at least 3 steps	82(9.9%)

Multiple responses possible

### Factors associated with BP/CR

Place of residency, current marital status, escorting wife to antenatal care in previous pregnancy, number of children, level of education, knowledge of at least one danger sign during pregnancy, knowledge of at least one danger sign during delivery and knowledge of at least one danger sign during the postpartum period were associated with BP/CR in the bivariate logistic regression analysis. Further analysis by using multivariable logistic regression demonstrates that completing college education have a significant positive association with the steps taken for birth preparation and complication readiness (AOR = 3.2, 95% CI: 2.8, 4.6). Being married, escorting wife to antenatal care, and urban residence were also found to be significantly associated with the steps taken for birth preparation and complication readiness. Furthermore, the steps made towards birth preparation and complication readiness were found to have significant associations with knowledge of at least one danger sign during pregnancy (AOR = 3.3, 95% CI: 3.1, 3.9), during delivery (AOR = 2.2, 95% CI: 1.1, 2.8) and post partum period (AOR = 1.8, 95% CI: 1.1, 2.4). (Table 4).

**Table 4:** Association between selected socio-demographic characteristics, obstetric characteristics, knowledge of danger signs and BP/CR

Characteristic	Prepared N (%)	Less prepared N (%)	Bivariate Analysis Unadjusted OR (95%CI)	Multivariable Analysis AOR (95%CI)
Age				
18–24	12(7.8%)	142(92.2%)	1	
25–34	15(7.6%)	183(92.4%)	0.4 (0.3–1.2)	
35–44	43(12.2%)	310(87.8%)	1.7 (0.6–6.2)	
> 44	12(10.1%)	107(89.9%)	0.5 (0.2–2.8)	
Number of children				
1–2	18(10.4%)	155(89.6%)	1	
3–4	39(9.7%)	364(90.3%)	0.4 (0.2–5.4)	
More than 4	25(10.1%)	223(89.9%)	1.4 (1.3–2.1)	
Education				
No education	9(6%)	141(94%)	1	
Primary Incomplete	11(7.9%)	127(92.1%)	1.5 (0.3–6.2)	
Primary complete	20(6.4%)	291(93.6%)	1.7 (0.8–3.9)	
Secondary	17(13.7%)	107(86.3%)	1.3 (1.2–2.5)	
College and above	25(24.7%)	76(75.3%)	3.8 (2.9–5.1)	3.2(2.8–4.6)*
Marital status				
Married	78(10.8%)	643(89.2%)	3.1 (2.8–3.6)	2.3(1.6–3.5)*
Single/divorced/widowed	4(3.8%)	99(96.2%)	1	
Residence				
Urban	46(24.6%)	141(75.4%)	5.8 (4.3–6.3)	4.2(2.9–5.6)*
Rural	36(5.7%)	601(94.3%)	1	
Escorted Wife to ANC in the previous pregnancy				
Yes	43(12.9%)	291(87.1%)	2.7 (1.8–3.2)	1.7(1.6–2.9)*
No	39 (8%)	451 (92%)	1	
Knowledge of at least one danger sign during pregnancy				
Yes	61(15)	346(85)	3.1 (2.5–3.4)	3.3(3.1–3.9)*
No	21(5.1)	396(94.9)	1	
Knowledge of at least one danger sign during labor				
Yes	39(14.4)	232(85.6)	3.7 (2.3–3.8)	2.2(1.1–2.8)*
No	43(7.8)	510(92.2)	1	
Knowledge of at least one danger sign postpartum				
Yes	37(17.4%)	176(82.6%)	2.6 (1.5–2.7)	1.8(1.1–2.4)*
No	45(7.4%)	566(92.6%)	1	

*p value< 0.05, in the table the number “1” indicates the reference variable

## Discussion

As part of a comprehensive antenatal care provision a pregnant woman and her family should be provided with detailed information about pregnancy, child birth, and obstetric danger signs that may occur in the process of child birth [4]. Antenatal care creates a good opportunity for the service providers to develop a birth plan with a women and her spouse. Involving men on the maternal health care system improves health service utilization, decrease rate of maternal depression, raise maternal self-esteem, and decreased possibility of childbirth complications [16–20].

Male residents in Northwest Ethiopia were found to have inadequate awareness regarding obstetric danger signs. This could be due to the low percentage of participants who had accompanied their spouses to antenatal care during previous pregnancy (40.5%) and low percentage of facility delivery rate (24.4%). It could also be due to deficiencies in community health education programs in the country. The findings of this research are similar with a study conducted in Southern Ethiopia which reported that only 42.2% of men were found to be aware of the obstetric danger signs [21]. Similarly, other studies conducted in different countries also reported men’s inadequate knowledge concerning the obstetric danger signs. However, our findings are in contrast with one study that assessed Kenyan men’s awareness of obstetric danger signs in which the median knowledge score was reported to be 9 out of 10 selected danger signs. This discrepancy may relate to differences in study method; in the Kenyan study men were asked to say yes or no after 10 danger signs were read to them by the interviewer [22].

Although majority of maternal morbidities and mortalities occur in the immediate postpartum period [23], men in this community were able to state fewer possible danger signs in the postpartum period as compared to other periods (ante partum and delivery). This may be due to low level of postnatal care utilization (20.2%) in communities of Northwest Ethiopia [24] and/or the cultural practice in rural parts of Ethiopia that postpartum women spend their postpartum period with their mothers. This result is in line with a study conducted in rural Tanzania that reported higher numbers of danger signs stated in the ante partum period [14].

We found discrepancies between men’s knowledge and the common causes of maternal complications. The most common obstetric danger signs men reported in relation to pregnancy were high fever (12.7%), severe abdominal pain (11.8%), and vaginal bleeding (11.4%). The finding of this study is in agreement with a study conducted in Southern Ethiopia that reported vaginal bleeding and severe abdominal pain as the most stated danger sign that may occur in the ante partum period [21]. In a Tanzanian study high fever, abdominal pain and vaginal bleeding were also commonly mentioned danger signs in the ante partum period [14]. A review conducted in 2014 reported that among all maternal deaths in Ethiopia obstructed labor and sepsis accounts for 29 and 13% respectively [15]. In a study conducted in Southwest Ethiopia, the incidence of obstructed labor was reported to be 12.2% [25]. However, prolonged labor as a danger sign during delivery was mentioned only by 10.3% of participants. Only 12% of participants mentioned fever as a danger sign in the postpartum period. Hence, awareness creation interventions are recommended in order to reduce major causes of maternal mortality and morbidity in this community.

Men’s involvement in birth preparedness and complication readiness in this community was found to be scarce. Only 82(9.9%) participants reported making at least three of the six assessed steps during the birth of their last baby. A southern Ethiopian study also reported a low level (9.4%) of male involvement in BP/CR issues [21]. Moreover, the finding of this study is also in line with studies from other countries like Tanzania and Uganda [14, 26]. The low level of involvement in BP/CR may be explained by the low antenatal service utilization in this area, the poor quality of the service, mostly husbands did not accompany their wives during antenatal visit and even if they come there are also failures from the service providers to invite husbands to join the counseling process [26–29].

Higher level of education, being married, escorting wife to antenatal care, urban residence were found to have positive significant association with birth preparation and complication readiness. This may be due to the fact that those who have higher level of education have good level of understanding of the complications and importance of having a birth plan. In addition to that urban dwellers will access information as well as birth kits easily as compared to rural dwellers. Most of the educated ones have urban residency which can also explain the low level of birth preparation and complication readiness among rural residents. Escorting wife to antenatal care will increase exposure with health professionals that may improve the probability of being prepared for complications and childbirth. These factors were also found to be associated with men’s involvement in BP/CR in studies conducted among other African countries like Tanzania and Uganda [30–32].

In the current study men’s level of involvement in BP/CR was found to have a significant association with knowledge of obstetric danger signs during pregnancy, labor and postpartum. This may be due to the fact that knowledge of danger signs leads to greater anticipation and preparation to lessen effects of pregnancy and childbirth complications by reducing the first two delays (delay in decision making and delay in transportation) to tackle obstetric complications. The norms and values of the community towards women may have affected their interest of engaging in the maternal health issues. This finding is similar with a study conducted among male residents of southern Ethiopia which reported that awareness of danger signs doubles involvement [21].

### Limitations and strengths of the study

As this is a cross sectional study, it is not possible to provide a causal relationship between being well-prepared and other variables. Moreover, the participants were required to remember things happened in the past 2 years so there is a possibility of recall bias. Although, all the necessary precautions were followed to maintain the quality of the data, it is not possible to avoid the effect of data collector’s attitude on the quality of the data. These limitations mean that caution is required when generalizing the results to other settings. Despite these limitations our study demonstrates a lot of strengths. One of the strengths of this study was the large number of participate in the study and men’s knowledge of danger signs and BP/CR were not assessed in this area previously.

## Conclusions and recommendations

The current study demonstrated men’s low level of knowledge concerning obstetrical danger signs as well as their involvement in birth preparedness and complication readiness in Northwest Ethiopia. This study also showed a strong association between BP/CR and men’s level of awareness concerning obstetric danger signs. Additionally, BP/CR was also found to have a significant association with higher educational level, being married, escorting wife to antenatal care in their spouses’ previous pregnancy, and being an urban resident. In patriarchal communities like Ethiopia so as to improve the maternal health service utilization, involving men in the maternal health care system without worsening the already existing gender inequalities is an essential strategy. Hence, it is recommended to advocate policies and strategies that can improve men’s level of awareness and their engagement in the maternal care through health education and incentives. Community based awareness creation by utilizing mass media and campaigning is recommended. It is recommended to give special emphasis to improving utilization of postnatal care to improve the low level of knowledge in the post partum period, were most of maternal mortalities occur, as compared to the ante-partum and intra-partum period.

## Additional file

Additional file 1: Questionnaire. (DOCX 2172 kb)

## Abbreviations

ANC: Antenatal care
BP/CR: Birth preparedness and complication readiness
