# Supplementary material for: Male involvement in the maternal health care system: implication towards decreasing the high burden of maternal mortality
Source: BMC Pregnancy Childbirth. 2018 Dec 14;18:493. doi: 10.1186/s12884-018-2139-9 (PMC6295014; doi:10.1186/s12884-018-2139-9)
Supplement: Supplementary file 1 — Questionnaire. (DOCX 2172 kb) [file 12884_2018_2139_MOESM1_ESM.docx]

**Cover letter**

**Consent Information Sheet**

My name is __________________________. I am conducting a research on **‘Male involvement in the maternal health care system: Implication towards decreasing the high burden of maternal mortality’.** The research project is approved by the Research Ethics committee of School of medicine, University of Gondar. Your participation is purely based on your willingness and you have the right to choose not to take part in this study. If you choose to take part, you have the right to stop at any time.

If you agree to participate in the study, you will be asked to answer some questions about yourself and your level of knowledge and involvement in maternal health services. The interview with you will take about 30 minutes. The information that you provide will be kept confidential by using only code numbers and locking the data.

Based on the understanding of the information I gave you, are you willing to participate in this study?

1. Yes
2. No

**Part 1: Socio -demographic characteristics**

| **Sr. No** | **Questions** | **Choice Answers** |
| --- | --- | --- |
| 101 | Age | in years: __________ |
| 102 | Did your wife give birth in the last 24 months, either to a baby that was born alive or a baby that was born dead? (If the answer is yes continue with other questions) | 1. Yes 2. No |
| 103 | What is your religion? | _______________ |
| 104 | What is your current marital status? | _______________ |
| 105 | Are you currently living with your partner? | 1. Yes 2. No |
| 106 | What is the highest grade you have completed? | _______________ |
| 107 | What is your job? | _______________ |
| 108 | How many children do you have? | _______________ |
| 109 | Where is your place of residence? | 1. Rural 2. Urban |

**Part 2: Spouse’s obstetric characteristics, Men’s knowledge of danger signs during pregnancy, labor and postpartum.**

| **Sr. No** | **Questions** | **Choice Answers** |
| --- | --- | --- |
| 201 | Did your wife had any obstetric complication in previous pregnancy? | 1. Yes 2. No |
| 202 | Escorted wife to ANC during her previous pregnancy? | 1. Yes 2. No |
| 203 | Where did she delivery her previous pregnancy? | 1. Home 2. Health facility |
| 204 | In your opinion, what are some serious health problems that can occur during pregnancy that could endanger the life of a pregnant woman? (You can state more than one problems) |  |
| 205 | In your opinion, what are some serious health problems that can occur during labor and childbirth that could endanger the life of a pregnant woman? (You can state more than one problems) |  |
| 206 | In your opinion, what are some serious health problems that can occur during the first 10 days after birth that could endanger the life of the woman? (You can state more than one problems) |  |

**Part 3: Birth preparedness and complication readiness (BP/CR) of men in previous pregnancy**

| **Sr. No** | **Questions** | **Choice Answers** |
| --- | --- | --- |
| 301 | Which one of the following things have you done while during your spouse’s last pregnancy? (You can tick more than one answers) | 1. Prepared a birth kit? 2. Arrangements for funds/finances, saved money? 3. Arrangements for transportation? 4. Arrangements for a blood donor? 5. Where she should give birth to your baby? 6. Arrangements for a healthcare professional to deliver your child? |

**Thank you for your participation!!!**
